# Reduced Connectivity in the Self-Processing Network of Schizophrenia Patients with Poor Insight

**DOI:** 10.1371/journal.pone.0042707

**Published:** 2012-08-09

**Authors:** Edith J. Liemburg, Lisette van der Meer, Marte Swart, Branislava Curcic-Blake, Richard Bruggeman, Henderikus Knegtering, André Aleman

**Affiliations:** 1 Department of Neuroscience, University Medical Center Groningen and BCN NeuroImaging Center, University of Groningen, Groningen, The Netherlands; 2 Rob Giel Research Center, University Medical Center Groningen, Groningen, The Netherlands; 3 Lentis, Center for Mental Healthcare, Groningen, The Netherlands; 4 Department of Psychology, University of Groningen, Groningen, The Netherlands; Hangzhou Normal University, China

## Abstract

Lack of insight (unawareness of illness) is a common and clinically relevant feature of schizophrenia. Reduced levels of self-referential processing have been proposed as a mechanism underlying poor insight. The default mode network (DMN) has been implicated as a key node in the circuit for self-referential processing. We hypothesized that during resting state the DMN network would show decreased connectivity in schizophrenia patients with poor insight compared to patients with good insight. Patients with schizophrenia were recruited from mental health care centers in the north of the Netherlands and categorized in groups having good insight (n = 25) or poor insight (n = 19). All subjects underwent a resting state fMRI scan. A healthy control group (n = 30) was used as a reference. Functional connectivity of the anterior and posterior part of the DMN, identified using Independent Component Analysis, was compared between groups. Patients with poor insight showed lower connectivity of the ACC within the anterior DMN component and precuneus within the posterior DMN component compared to patients with good insight. Connectivity between the anterior and posterior part of the DMN was lower in patients than controls, and qualitatively different between the good and poor insight patient groups. As predicted, subjects with poor insight in psychosis showed decreased connectivity in DMN regions implicated in self-referential processing, although this concerned only part of the network. This finding is compatible with theories implying a role of reduced self-referential processing as a mechanism contributing to poor insight.

## Introduction

Patients with schizophrenia often have difficulties with social and emotional cognitive processing [1], [2], including self-reflective processes [3]. Such impairments may have important consequences for successful functioning in a social community [1], [4]. Self-referential processing deficits, which may already be present before the onset of the disorder, have been proposed to underlie these social and emotional deficits as well as first rank schizophrenic symptoms, e.g. [5]–[10]. Such self-related processing deficits may include the formation and maintenance of an accurate representation of one’s traits, abilities and attitudes, or self-reflection [11], [12]. This self-reflective processing is essential in the evaluation of one’s personal behavior as well as in interpersonal communication [2]. More specifically, it has been proposed that self-reflective processing may underlie poor illness insight in patients with schizophrenia [12]–[14].

Impaired insight has been considered to be a core feature of schizophrenia [15]. Poor insight in schizophrenia has been associated with poorer global functioning [16]–[18], greater severity of psychopathology [19], increased relapses and hospitalizations, poorer long term prognosis [20] and reduced treatment compliance [21], [22]. Interestingly, lack of insight in schizophrenia appears to be self-specific, as most patients recognize symptoms in other patients, but fail to do so in themselves [23], [24]. This implies that lack of insight may be caused by disturbed abilities of self-referential processing [12]. Thus, studying the neural link between insight and self-referential processing may reveal important clues with regard to the underlying deficit in patients lacking insight. If patients with schizophrenia have attenuated capacities to reflect on their situation and on other self-relevant information, this could be a barrier for obtaining insight that one suffers from a severe psychiatric disorder.

In terms of brain regions that underlie self-referential processing, research points towards a set of medial brain areas comprising the posterior cingulate cortex (PCC), anterior cingulate cortex (ACC) and the dorsomedial and ventromedial prefrontal cortex (d & vMPFC) [25], [26], together referred to as the cortical midline structures (CMS) [11], [12], [27]. In patients with traumatic brain injury in the CMS [28], patients with mild cognitive impairment [23] and schizophrenia patients [29]–[32], an association between impaired insight and decreased activation of medial frontal and other CMS regions has been demonstrated.

The CMS show a large overlap with the so-called default mode network (DMN). This is a network of brain areas that is active during rest [33], [34] and involved in processing information related to the self [33], [35]. The brain areas in the network show synchronized slow fluctuations (<0.1 Hz) in the BOLD signal [36], [37]. Areas in this network include the ventral and dorsal medial prefrontal cortex (vMPFC and dMPFC), anterior cingulate (ACC), the posterior cingulate (PCC)/retrosplenial cortex (RspC) and adjacent precuneus, inferior parietal lobule (IPL), medial temporal cortex (MTG) and hippocampal formation [34], [36]. The default mode network appears to encompass subnetworks with distinct functions [34], consisting of an anterior part (ACC/MPFC), a posterior part (PCC, precuneus and IPL), and possibly a ventral part with temporal and ventral prefrontal regions [11], [34]. Studies in schizophrenia patients have found disturbances in DMN structures, with mainly lower medial PFC connectivity compared to healthy controls [38]–[41] but also altered connectivity within posterior DMN areas, disturbed prefrontal-parietal communication [32], [41]–[48], or reduced connectivity between other DMN regions [42]–[45], [48]–[50]. Of note, some studies showed increased frontal connectivity [51]–[53].

Structural MRI studies have related poor insight in schizophrenia patients to decreased volume of prefrontal and other DMN regions [54], [55], which may be related to poor self-monitoring [56]–[59]. Patients or people at risk for psychosis indeed show altered brain activation during self-reflection and theory of mind [29], [30], [32], [60]. Moreover, review studies have shown that schizophrenia patients have a decreased prefrontal and posterior DMN activation in resting state studies [31], [61]. Finally, decreased white matter integrity between DMN areas was also related to poor insight [62]. No studies have as yet investigated resting state connectivity in relationship to poor insight in psychosis.

Connectivity analysis may further the understanding of neural systems beyond the task-activation fMRI designs [63], [64]. Resting state BOLD fluctuations may reflect spontaneous neural activity as most resting state patterns overlap with known brain networks [64], [65], and they may even predict an individual’s task performance or behavior [63]. Moreover, their functional connectivity follows the anatomical outline of white matter bundles [64]. Whereas task-based activation can provide information about the function of separate brain areas, functional connectivity may thus provide information about interaction of brain areas [64]. Resting state research of the DMN is especially interesting with regard to the issue of insight, because we expect a relation between the key function of the DMN, namely self-referential processing, and insight.

Studying resting state fluctuations may have some advantages over task-based fMRI. Experimental control of differences in task performance between groups is not necessary and relatively ill patients groups with limited capacities can be investigated [65], [66]. Only intrinsic differences of the brain, and not differences in cognitive abilities, will explain differences in connectivity. Moreover, resting state functional connectivity may be a more natural, ecologically relevant, measure of brain activation than task-based fMRI [33] as it reflects intrinsic brain interactions [67].

Independent component analysis (ICA) [68] can separate the fMRI signal into spatially independent networks that show shared temporal fluctuations [67], [68]. Independent components (i.e. networks) contain brain areas that show similar fluctuations and are assumed to be functionally linked. The size and strength of the identified networks (components) may differ between individuals and groups sharing a specific trait [67], [68], as may cooperation between different networks [69]. In this study, we will focus on the DMN because this has been related to self-related processing [35]. We expect to identify an anterior and posterior DMN subnetwork as described earlier, as these have been identified previously using ICA [69], [70].

We hypothesize that schizophrenia patients with poor insight may show impaired connectivity of the DMN during rest, which may reflect attenuated self-related processing associated with decreased awareness of symptoms [27]. We therefore compared connectivity of brain areas within anterior and posterior DMN components to the other parts of that component between patients with good and with poor insight. A healthy control group was used as a reference. Moreover, we conducted a group comparison of connectivity strength between the anterior and posterior DMN components, as we hypothesize that impaired connectivity between the anterior and posterior DMN may also contribute to impaired insight.

## Methods

### Ethics Statement

The study was approved by the local medical ethical committee (Medische Ethische Toetsingscommissie van het Universitair Medisch Centrum Groningen) according to the declaration of Helsinki. All subjects gave oral and written informed consent after the study procedure had been fully explained. All subjects ware capable of signing the informed consent as they were able to live independent, no permanent inpatients, had no care givers taking over responsibilities from them, and all allowed to sign informed consent themselves. All subject data was handled anonymously.

### Study Population

The study sample included 44 patients with schizophrenia. Patients were recruited from mental health care centers in the north of the Netherlands, three or four patients came from western parts of the Netherlands. Patients were participants in an fMRI study on neural correlates of auditory hallucinations or a study on cognitive emotional processing; in both studies a resting state scan was part of the research protocol. Diagnosis of schizophrenia according to DSM-IV criteria was confirmed with the SCAN 2.1 diagnostic interview [71]. A healthy control group matched to the patients on age, gender, handedness, and education level was included. This group was included to deduce whether patients showed similar DMN properties as healthy subjects. Healthy controls were excluded in case of psychiatric history, which was confirmed with the screenings questions of the SCAN 2.1 interview. For subject characteristics, see Table 1. Patients were asked to give an overview of the medication they were taking at the moment. The patients reported to use the following medication; antipsychotics: aripiprazole (9x), chlorprotixene (1x), clozapine (15x), haloperidol (4x), olanzapine (9x), paliperidone (1x), penfluridole (1x), perphenazine (1x), pimozide (1x), pipamperone (1x), quetiapine (7x), risperidone (10x), sulpiride (1x), and zuclopentixole (2x); antidepressants: amytriptyline (1x), bupropione (1x), citalopram (3x), clomipramine (1x), fluoxetine (2x), fluvoxamine (1x), mirtazapine (1x), paroxetine (2x), nortriptylin (1x), trazodone (1x), and venlafaxine (2x); benzodiazepines: diazepam (3x), flurazepam (1x), lorazepam (3x), oxazepam (7x), temazepam (5x); other: atenolol (1x), biperiden (6x), carbamazepine (1x), lithiumcarbonate (6x), pantaprazol (2x), promethazine (1x), valproic acid (1x).

**Table 1:** Demographical data.

	Good insight (n = 25)		Poor insight (n = 19)		Controls (n = 30)		Statistical test score (Z or X^2^)	p-value
Mean age (SD)	33.4	(11.2)	35.9	(11.9)	33.4	(10.5)	.69	.71
Mean education (SD)	3.52	(1.3)	3.53	(1.2)	4.1	(1.1)	1.1	0.59
Gender (M/F)	9/16		7/12			15/15	.0	0.51
Handedness (L/R)	3/22		2/17			6/24	.0	1.0
PANSS G12 (SD)	1.3	.5	3.7	.8			5.9	<.005
PANSS Positive (SD)	14.3	(4.8)	17.1	(4.8)			1.96	.050
PANSS Negative (SD)	14.3	(4.3)	14.4	(4.8)			.21	.83
PANSS General -12 (SD)	25.8	(8.3)	28.1	(7.4)			1.34	.18
Illness duration years (SD)	10.5	9.6	8.9	8.2			.46	.67
No antipsychotic (%)	0		21.1				6.1	.11
Typical (%)	8.0		10.5					
Atypical (%)	68.0		47.4					
Typical + atypical (%)	2.0		10.5					

Overview of demographical data of the good insight and poor insight groups and the control group; The PANSS general item is shown without item G12. The fifth column shows the Z (Mann-Whitney) or Chi-square (Kruskal-Wallis and Chi-square test for independence) values of the statistical comparisons and the fifth the p-values.

### Measures

The most important measure of the study was connectivity of brain areas within the anterior and posterior DMN component to the rest of that component. Differences in connectivity within a component were compared between groups by doing a voxel-wise group comparison of the spatial maps of individual subjects. Connectivity between components was also determined by correlating the time courses of the anterior and posterior DMN component. These were converted to Z-scores and compared between groups.

### Design

The primary goal was to compare connectivity measures between patients with good and poor insight. A matched healthy control group was used as a reference. If possible, differences were statistically compared, but as described below, in some cases only qualitative comparison was possible.

All schizophrenia patients were interviewed with the Positive and Negative Syndrome Scale (PANSS) [72]. The PANSS interview measures three domains of symptoms, namely positive and negative symptoms and general pathology. Each item can be rated from 1 (not present) –7 (extreme). The interviews were performed by experienced and trained raters. Based on the rating of the interview item that measures illness insight (G12), patients were categorized into two groups with good insight (score 1–2, which are in the normal range) or poor insight (>2). Even though this is only one single item, strong correlations with more thorough measures of insight such as the Scale to Assess Insight (SAI; r = 0.88), Scale to Assess Insight – Expanded (SAI-E; r = 0.90), or the Insight and Treatment Attitudes Questionnaire (ITAQ; r = 0.90) have been demonstrated [73], [74], confirming that the PANSS G12 item reliably rating insight.

Education level was rated according to a six point scale defined by Verhage [75], which ranges from primary school (1) to university level (6). Handedness was confirmed by the Edinburgh handedness inventory [76]. Age and education level were compared between controls and the two patients groups with a Kruskal-Wallis H test (α<0.05). Between patient group differences in PANSS subscales were tested with a Mann-Whitney U test. For the PANSS General pathology subscale the Insight item G12 was subtracted from the total score, because this item was a selection criterion for both groups. A Chi-square test for independence (α<0.05) was used to test for differences in gender and handedness. All statistical tests were performed with Statistical Package for Social Sciences (SPSS) 16. Exclusion criteria for the study consisted of MRI incompatible implants, possible pregnancy, claustrophobia and non-native Dutch speakers.

### MRI Procedure

All subjects underwent a resting state fMRI scan. They were instructed to close their eyes, relax, and to stay awake. Subjects were reminded of this just before the scan started. A 3 T Philips Intera MRI scanner (Best, The Netherlands) equipped with a 8-channel SENSE head coil was used to acquire 200 whole brain echo-planar functional images (EPÌs), TR 2.3 s and TE 28 ms. The volumes contained 39 (old sequence) or 43 (after scanner upgrade) interleaved slices (3.8×3.8×3 mm) with a 0 mm slice gap and a 85° flip-angle (FOV = 220×117×220 mm). The duration of the scan was 460 seconds. A high-resolution, transverse T1 anatomical was also acquired for overlay of statistic images (160 slices; voxel size 1×1×1 mm; FOV 256×220×256 mm).

### Analysis

The raw images were converted to ANALYZE format and analyzed using Statistical Parametric Mapping (SPM8; FIL Wellcome Department of Imaging Neuroscience, London, UK) running on Matlab 7.1. Images were first corrected for slice-time differences and realigned to the first functional image. The mean image created during realignment was co-registered to the anatomy, together with the functional images, and the anatomy and functional images were normalized (voxel size 3×3×3 mm) to the T1 template of SPM. Finally, images were smoothed with a 10 mm FWHM isotropic Gaussian kernel. Additional filtering was not necessary, because artifacts will generally represented by separate components in ICA [67], [68].

After the preprocessing, images were processed in Group ICA FMRI Toolbox (GIFT; http://icatb.sourceforge.net/gift/gift_startup.php) [68]. For referential purposes, a separate ICA was conducted on the group of healthy control subjects. Healthy subjects were not included in the ICA of patients but treated separately, because subtle differences in spatial maps of patients, only distinguished based on insight score, may disappear due to inclusion of a group with different network properties, such as healthy controls [68].

The mean number of independent components (I
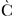
s) was estimated using Maximum Description Length (MDL) and Akaike’s criteria [77], to prevent splitting or merging of components [65]. Images were intensity normalized before ICA estimation, which implied scaling the time courses to a mean of 100. The intensity normalized images (patients and controls separately) were decomposed into a set of spatially independent components (for every subject) by the Infomax algorithm. A component consists of a time course showing the temporal fluctuations of that component, and a spatial map that shows the contribution of every voxel to that component. Stability of the components, i.e. whether a component has the tendency to split or merge with another component, was validated by running the ICASSO toolbox implemented in GIFT using twenty iterations with both random iterations and bootstrapping [78].

Selection of the components of interest for both healthy controls and patients, namely the anterior DMN (including the ACC/MPFC) and posterior DMN (PCC/precuneus/IPL), was done by selecting components showing a large spatial overlap with a priori defined anatomical masks. Thus, the spatial component could also involve other brain areas, but involvement of the areas defined by the masks was crucial. These anatomical masks of the ACC/MPFC (to select the anterior DMN component) and of the PCC/precuneus (for posterior DMN component selection) were created with WFU–pickatlas (http://www.nitrc.org/projects/wfu_pickatlas). Masks provided by WFU pickatlas are based on brain regions defined by Talairach and Tournoux (1998) that were implemented in this toolbox after conversion to MNI space [79], [80].

Spatial maps of selected anterior and posterior DMN components were visually compared between patients and controls to establish whether similar networks were present in both groups. Statistical comparison of image maps of two different ICÀs is unjustified, because the outline of image maps may differ between groups due to the separate ICA unmixing procedure of the image time courses in both groups.

After that, for the patients the reconstructed individual spatial maps of the anterior and posterior DMN component were entered in a two sample t-test random-effects analysis comparing the good versus poor insight group. This analysis shows brain areas that are differently connected to the rest of the anterior or posterior DMN component. A statistical threshold was applied of p<0.001, as has been done previously [83]. The analysis was restricted to areas that significantly contributed to the ICA component, as previously described by [70]. This was done because ICA components maps have values close to zero in areas where the time course of that component is not represented. Voxel intensities in these areas are mainly determined by noise properties and may in group comparison lead to false-positive clusters. Since we formulated a specific hypothesis comprising specific brain areas and used a mask to restrict the search volume, and because a comparison between two groups of patients was performed, cluster correction was not applied to avoid type II errors [81]. In an additional analysis, a voxel-wise linear regression was performed with the time courses of each voxel in the component maps of the DMN against the PANSS G12 Insight scores.

In another analysis, a correlation was calculated between the anterior and posterior DMN component time courses of all subjects. The correlations were converted to Z-scores by a Fische
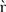
s Z transformation with *Z* = ½*ln((1+*r*)/(1−*r*)),where *r* represents the correlation. These data were loaded in SPSS. The Z-scores between the time courses of the anterior and posterior DMN of all patients were compared to those of controls and the Z-scores of patients with poor insight to those of patients with good insight using Mann-Whitney U tests (α = 0.05).

Two additional analyses were performed. First, because there was a significant difference in the PANSS positive subscale between groups, this subscale was added as a covariate to the group comparison. Second, as DMN regions have been shown to deactivate during task-performance, we also investigated whether the regions that we identified in the ICA group comparison overlapped with regions that showed task-related deactivation. For this, we analyzed a language task involving valence evaluation (positive, negative) of visually presented words that was performed by subjects during scanning. Deactivation of the DMN during task performance was shown by contrasting the fixation cross of the task with task blocks. The clusters showing a difference in DMN connectivity between the good and poor group were then overlayed on the task-related deactivation (Figure S1).

## Results

Twenty five patients were classified as having good insight, and nineteen patients were classified as having poor insight. The demographical characteristics of these two groups were compared, also with respect to the controls when applicable (see Table 1). The PANSS Positive subscale was significantly different between groups, but there was a significant correlation between PANSS G12 and the Positive symptom subscale (*r* = .36; *p* = .015), implying that patients with more positive symptoms had poorer insight. Therefore, the Positive symptom subscale was added as a covariate in the group comparisons, but this did not change the results. There was no significant difference in age, gender, handedness, education level and most PANSS scores, though the PANSS Positive subscale was significant.

**Figure 1:**
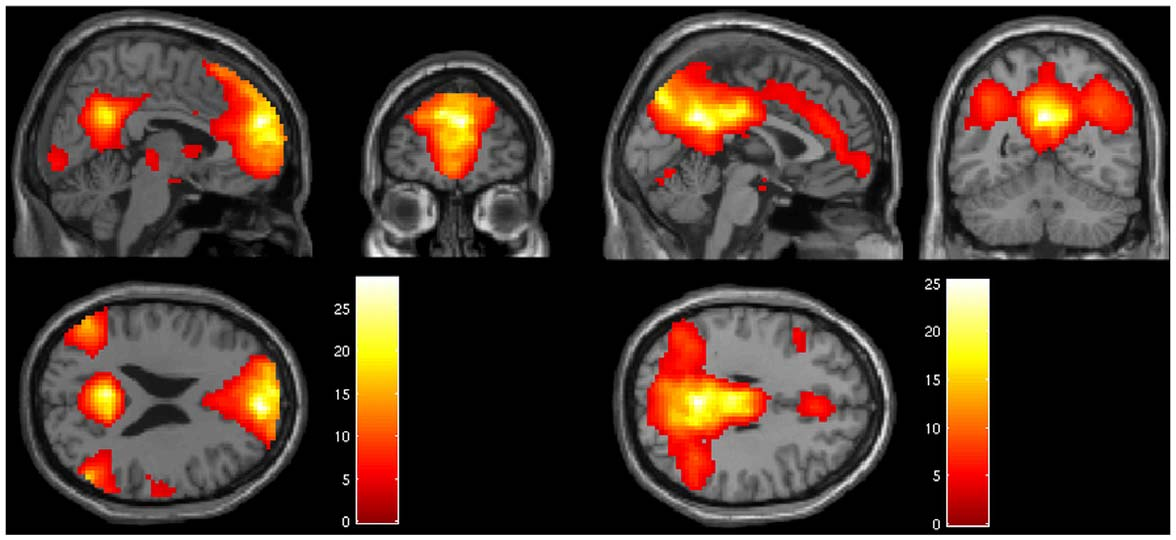
DMN in healthy controls. Components map of the DMN of healthy controls showing the anterior DMN on the left and the posterior part on the right (p<.001; k >10).

**Figure 2:**
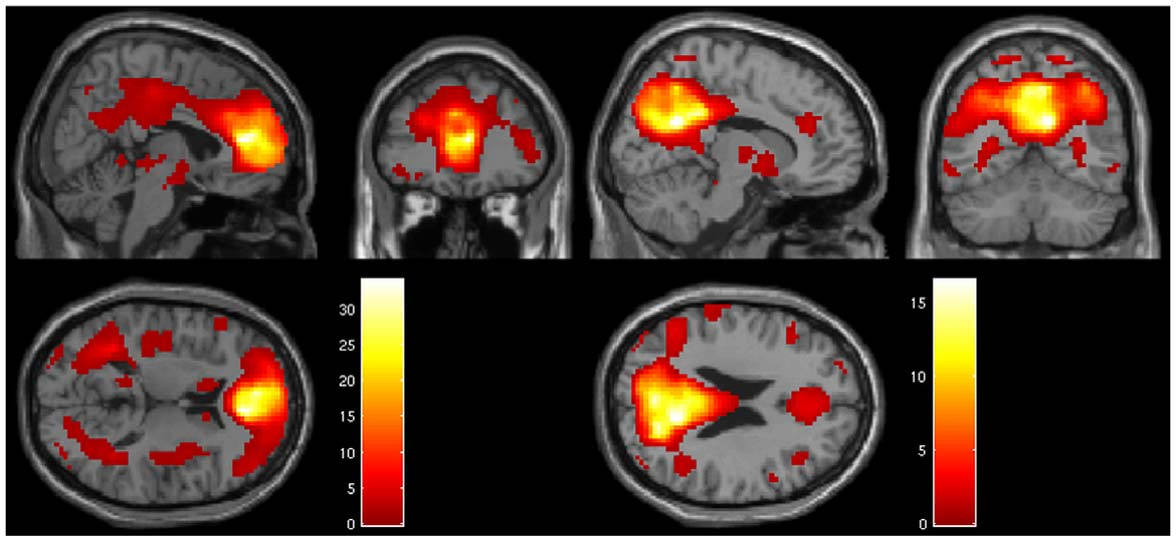
DMN in schizophrenia patients. Components map of the DMN of patients controls showing the anterior DMN on the left and the posterior part on the right (p<.001; k >10).

The component estimation resulted in an estimate of 32 components for the patients, and 30 for the healthy controls. The identified anterior default mode component encompassing the ACC/MPFC showed a spatial overlap correlation with the anatomical mask created by WFU Pickatlas of 21% for healthy controls (left side Figure 1) and of 56% for patients (left side Figure 2). Overlap of other components was <10%, indicating that the components of interest (anterior and posterior DMN) could be identified with high specificity. Visual inspection showed that the component map of the healthy controls had a more extended and stronger network contribution than the patients. The posterior component showed an overlap of 31% for healthy controls (right side Figure 1), and of 57% for patients (right side Figure 2).

**Figure 3:**
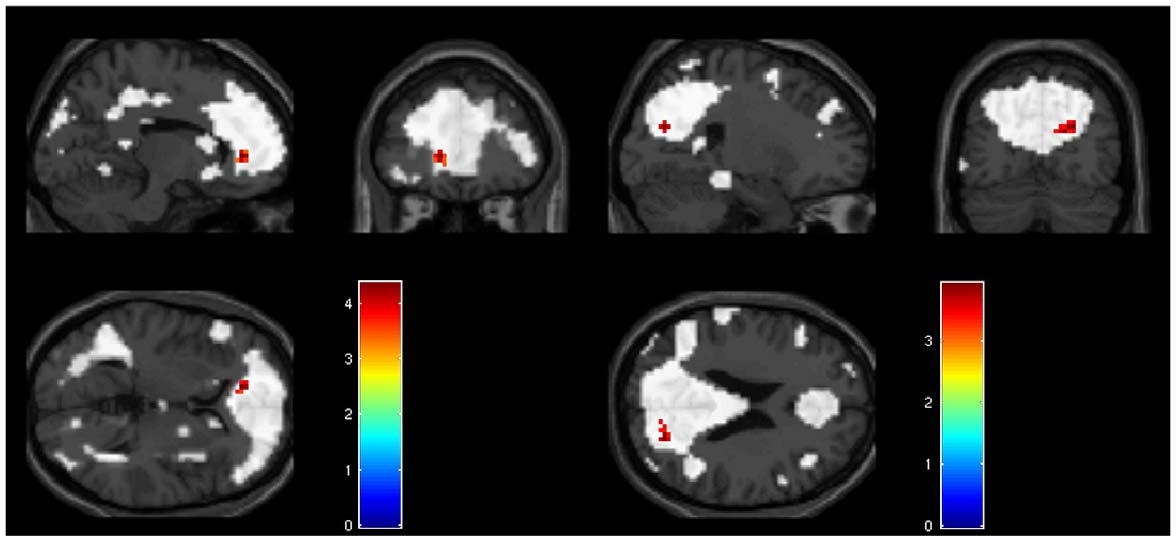
Differences in DMN connectivity between patients with good an poor insight. Group comparison of good vs. poor insight patients with the anterior component on the left showing the ACC, and the posterior component on the right showing the precuneus (p<0.001; k>10; masked with component image map).

The anterior and posterior components were compared with a two-sample t-test. Patients with good insight showed stronger connectivity of the ACC to the rest of the anterior DMN component compared to patients with poor insight: *t* = 4.37, *Z* = 3.94, cluster size = 18, *p*<0.001, xyz = −12 39 3 (Figure 3, left side). Subsequently, a voxel-wise regression between the image maps and the insight score was calculated. This revealed a cluster in the same location. In the two sample t-test of the posterior DMN component, a significant cluster was identified in the precuneus (*t* = 3.94, *Z* = 3.62, cluster size = 20, *p*<0.001, xyz = 24 −72 24, see Figure 3, right side). The linear regression with insight score resulted in the same cluster. There was no significant cluster in the poor vs. good insight t-test comparison for both components.

**Figure 4:**
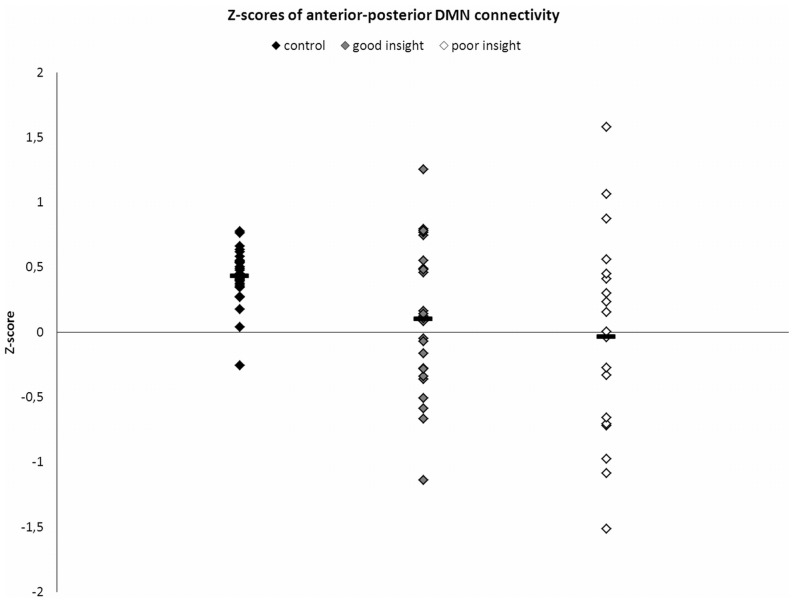
Connectivity between the anterior and posterior DMN in patients and controls. Z-scores of connectivity between the anterior and posterior DMN for healthy controls and schizophrenia patients with good and poor insight.

A correlation between the time courses of the anterior and posterior DMN component was calculated and converted to Z-scores. These Z-scores were compared between healthy controls and all patients, and between patients with good and poor insight. Z-scores are plotted per group in Figure 4. Whereas the Z-scores for the healthy controls were all above zero (with the exception for one subject), part of the patients showed a negative Z-scores with an overall mean around zero and a larger variation (SD_controls_ = 0.20, SD_patients_ = 0.66). This difference was significant (*U* = 388, *z* = −3.0, *p* = 0.003). Patients with poor insight showed the largest variation in Z-scores (SD_good insight_ = 0.56, SD_poor insight_ = 0.79), but did not differ significantly from the patients with good insight (*U* = 214, *z* = −0.56, *p* = 0.58).

Finally, adding the PANSS positive symptoms subscale as a covariate to the group comparison between good and poor insight did not change the results. In addition, during the valence evaluation task deactivation of DMN regions was observed. The clusters that differed significantly between good and poor insight groups overlapped with the DMN regions showing significant deactivation during the task (Figure S1).

## Discussion

In this study, the relationship between insight (awareness of illness) in schizophrenia and functional connectivity of regions in the default mode network (DMN) was investigated in patients with schizophrenia. The DMN connectivity pattern of patients clearly overlapped with the network in healthy control subjects, though the network was less extended (in accordance with e.g. [31], [46], [48]–[50], [61]. Importantly, patients with poor insight showed a lower connectivity within the anterior cingulate and precuneus compared to patients with good insight. Group differences were found in DMN regions that indeed deactivated during task performance, supporting our interpretation. Moreover, although the poor insight group showed significantly more positive symptoms, these did not explain the group differences Connectivity between anterior and posterior DMN was lower in all patients compared to controls, but there was no significant difference between patients with good and poor insight.

The result of reduced connectivity in the precuneus and ACC of the DMN in poor insight patients was in accordance with our expectations that poor insight would be related to decreased DMN connectivity [12], [28], [31], [35], although it may only concern part of the network. Studies assessing the overlap between self-referential processing and DMN activation, demonstrated that the ACC was consistently activated [82], [83] and thus seems to be particularly important for self-referential thought. Lesion studies demonstrated that lesions in/around this area can result in a diminished self-referential processing [84] and in a dysfunction of emotional self-control [85]. This suggests that reduced connectivity in this region may indeed result in abnormal self-referential processing. Whereas the ACC may be specifically involved in self-related processing, research has shown that precuneus activation is less self-specific and also activates during thinking about other persons [12], [82], [83]. Instead, the precuneus has been hypothesized to be involved autobiographical and episodic memory retrieval and mentalizing, which has been confirmed by several studies [12], [31], [35], [86], [87]. Consistent with this, structural neuro-imaging results point towards a relationship between impaired insight and reduced grey [58], [59] and white matter [62] in this region among others. Taken together, this suggests that hampered self-processing through a lack of integration of self-related information may underlie impaired insight in schizophrenia [12].

Schizophrenia patients had a lower, i.e. more negative, correlation between time courses of the anterior and posterior DMN. Though the mean connectivity was not significantly lower in patients with poor insight compared to good insight, the variation appeared to be higher in patients with poor insight. Disturbed connectivity between the frontal and posterior DMN could possibly have a modulating effect on insight. Patients with schizophrenia have shown decreased connectivity between the medial frontal cortex and other brain regions during self-reflective processing [32], [88]. Reduced communication between self-reflection areas may result in less transfer of self-related information (i.e. autobiographical or interoceptive information) of posterior areas to the anterior self-reflective areas.

One limitation of the study may be that insight was rated based on one item of a standardized interview. However, as we discussed above, this G12 item correlates highly with other more thorough measures of insight, suggesting that it can adequately index insight. Furthermore, it can be argued that subjects were not involved in self-reflective processing during resting state conditions. However, other studies have shown that self-referential processing is one of the major processes taking place during resting state [34], [35], [89], [90]. And as this is spontaneous self-referential processing, it was exactly the type of processing we were interested in. More research is needed to elucidate the contribution of different cortical midline structures in more detail.

In conclusion, schizophrenia patients with relatively preserved insight showed stronger connectivity than patients with poor insight in the anterior cingulate cortex and precuneus, both key regions in self-reflective processing. These findings tentatively support the hypothesis that poor insight may be related to impaired self-related processing.

## Supporting Information

Figure S1 **Group comparison of good vs. poor insight patients overlayed on task related deactivation.** The anterior component shown on the left with the increased ACC connectivity in the good insight group, and the posterior component on the right with the increased precuneus connectivity (p<0.001; k >10; masked with component image map), the task-related deactivation was defined by contrasting the fixation cross of a language task with task blocks.
(TIF)
