# Supplementary figures and images for: Reduced Connectivity in the Self-Processing Network of Schizophrenia Patients with Poor Insight
Source: PLoS One. 2012 Aug 9;7(8):e42707. doi: 10.1371/journal.pone.0042707 (PMC3415395; doi:10.1371/journal.pone.0042707)

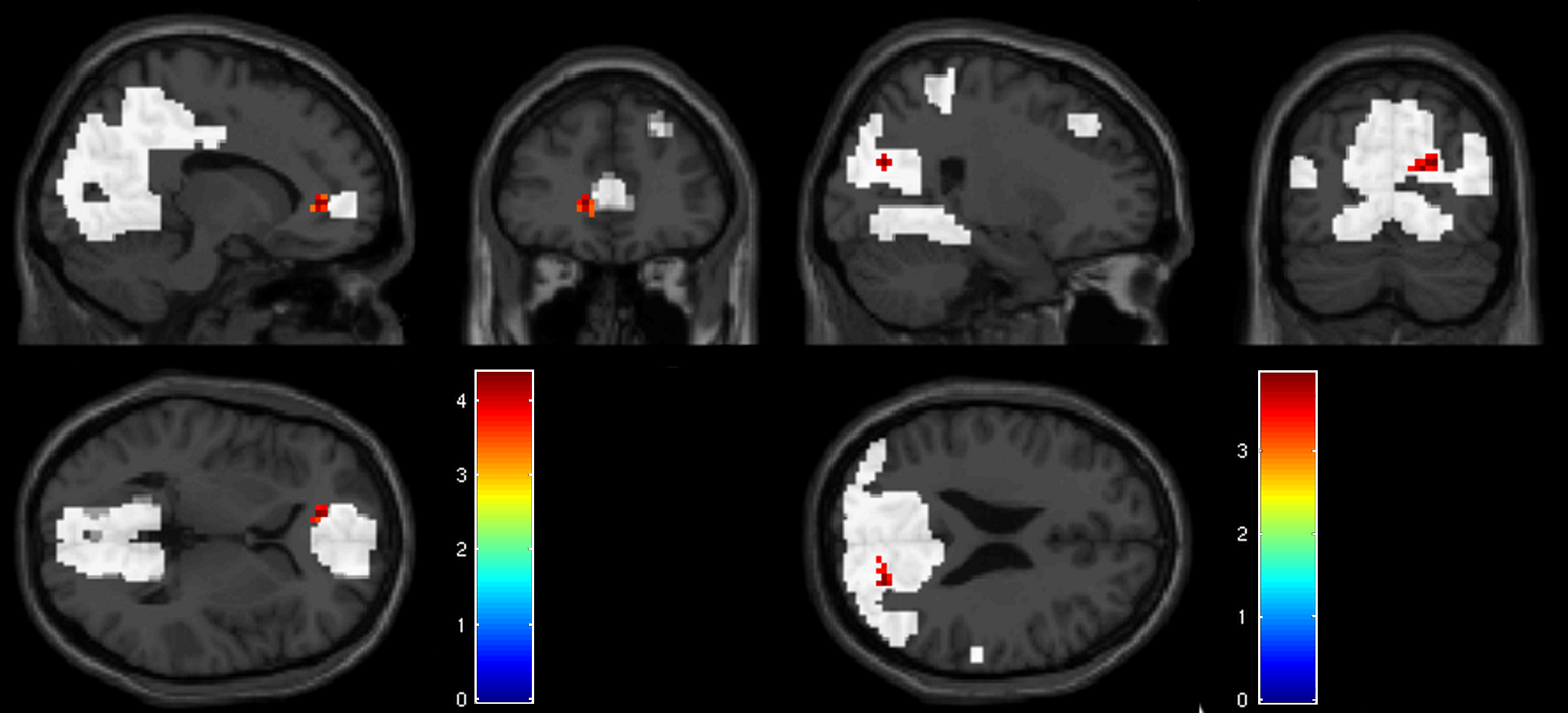

Supplement: Figure S1 — Group comparison of good vs. poor insight patients overlayed on task related deactivation. The anterior component shown on the left with the increased ACC connectivity in the good insight group, and the posterior component on the right with the increased precuneus connectivity (p<0.001; k >10; masked with component image map), the task-related deactivation was defined by contrasting the fixation cross of a language task with task blocks. (TIF) [file pone.0042707.s001.tif]
